# Ethyl 4-[(3,5-di-*tert*-butyl-2-hy­droxy­benz­yl)amino]­benzoate

**DOI:** 10.1107/S1600536810040742

**Published:** 2010-10-23

**Authors:** Raied Mustafa Shakir, Azhar Ariffin, Seik Weng Ng

**Affiliations:** a Department of Chemistry, University of Malaya, 50603 Kuala Lumpur, Malaysia

## Abstract

The title amine, C_24_H_33_NO_3_, has two substituted aromatic rings at either end of the –CH_2_NH– linkage [C_ar­yl_–CH_2_–NH–C_ar­yl_ torsion angle = 77.4 (3)°]. The amino and hy­droxy groups are hydrogen-bond donors to the carbonyl O atom of an adjacent mol­ecule. These hydrogen bonds give rise to a chain that runs along the *b* axis. One of the *tert*-butyl groups is disordered over two positions with a site-occupation factor of 0.834 (6) for the major occupied site.

## Related literature

For the structure of the Schiff-base reactant, see: Shakir *et al.* (2010 ▶).
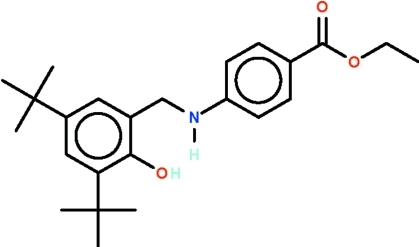


## Experimental

### 

#### Crystal data

C_24_H_33_NO_3_
*M* *_r_* = 383.51
Monoclinic, 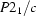

*a* = 17.788 (3) Å
*b* = 8.9872 (14) Å
*c* = 14.235 (2) Å
β = 101.414 (2)°
*V* = 2230.7 (6) Å^3^
*Z* = 4
Mo *K*α radiation
μ = 0.07 mm^−1^
*T* = 100 K
0.30 × 0.10 × 0.03 mm

#### Data collection

Bruker SMART APEX diffractometer
17094 measured reflections
3928 independent reflections
2428 reflections with *I* > 2σ(*I*)
*R*_int_ = 0.092

#### Refinement

*R*[*F* ^2^ > 2σ(*F* ^2^)] = 0.058
*wR*(*F* ^2^) = 0.154
*S* = 1.03
3928 reflections
283 parameters
45 restraints
H-atom parameters constrained
Δρ_max_ = 0.24 e Å^−3^
Δρ_min_ = −0.23 e Å^−3^

### 

Data collection: *APEX2* (Bruker, 2009 ▶); cell refinement: *SAINT* (Bruker, 2009 ▶); data reduction: *SAINT*; program(s) used to solve structure: *SHELXS97* (Sheldrick, 2008 ▶); program(s) used to refine structure: *SHELXL97* (Sheldrick, 2008 ▶); molecular graphics: *X-SEED* (Barbour, 2001 ▶); software used to prepare material for publication: *publCIF* (Westrip, 2010 ▶).

## Supplementary Material

Crystal structure: contains datablocks global, I. DOI: 10.1107/S1600536810040742/bt5371sup1.cif

Structure factors: contains datablocks I. DOI: 10.1107/S1600536810040742/bt5371Isup2.hkl

Additional supplementary materials:  crystallographic information; 3D view; checkCIF report

## Figures and Tables

**Table 1:** Hydrogen-bond geometry (Å, °)

*D*—H⋯*A*	*D*—H	H⋯*A*	*D*⋯*A*	*D*—H⋯*A*
O1—H1o⋯O2^i^	0.84	2.13	2.909 (3)	154
N1—H1n⋯O2^i^	0.88	2.07	2.827 (3)	143

Symmetry code: (i) 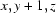.

**Fig. 1.**
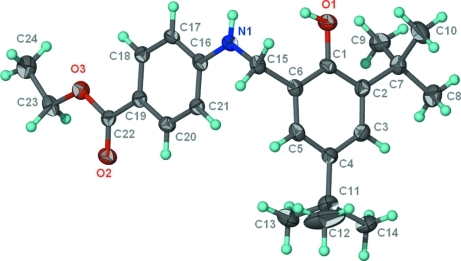
Anisotropic displacement ellipsoid plot (Barbour, 2001) of C24H33NO3 at the 70% probability level; hydrogen atoms are drawn as spheres of arbitrary radius. The disorder in one of the tert-butyl groups is not shown.

**Fig. 2.**
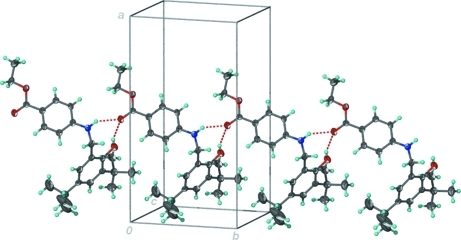
Hydrogen-bonded chain motif.

## supplementary crystallographic information

### Comment

The Schiff base, ethyl 4-(di-3,5-*tert*-butyl-2-hydroxybenzylideneamino)benzoate, is an intensely orange-colored compound whose color can be attributed to the azomethine double-bond; in the crystal structure, the two aromatic rings are aligned at 24.9 (1) ° (Shakir *et al.*, 2010). The reduction of this compound affords the title colorless compound (Scheme I, Fig. 1), which is a secondary amine. The amino and hydroxy groups are hydrogen-bond donors to the carbonyl oxygen atom of an adjacent molecule; the hydrogen bonds give rise to a chain that runs along the *b*-axis. One of the *tert*-butyl groups is disordered over two positions with respect to the methyl units in an 83.6 (6):16.4 (6) ratio.

### Experimental

Ethyl 4-aminobenzoate (0.35 g) dissolved in ethanol (5 ml) was added to 3,5-di-*tert*-butyl-2-hydroxybenzaldehyde (0.5 g) dissolved in ethanol (20 ml). Several drops of acetic acid were added. The solution was heated for 3 h. The solvent was evaporated and the product recrystallized from ethanol to yield orange prisms in 80% yield. The compound (0.5 g) was dissolved in methanol-THF (1:1) (20 ml). The solution was cooled to 273 K and three equivalents of sodium tetraborate were added. The mixture was stirred for 4 h until the orange color was discharged. The mixture was filtered and the solvent evaporated. The colorless compound was recrystallized from a hexane-ethanol mixure (yield 90%).

### Refinement

Carbon-bound H-atoms were placed in calculated positions (C—H 0.95–0.99 Å) and were included in the refinement in the riding model approximation, with *U*(H) set to 1.2–15*U*(C).

The amino and hydroxy H-atoms were also placed in calculated positions and their displacement parameters were also similarly tied (N–H 0.88 Å and O–H 0.84 Å).

One of the *tert*-butyl groups is disordered over two positions with respect to the methyl units in an 83.4 (6):16.6 (6) ratio; the C–C_methyl_ distances were restrained to 1.50±0.01 Å; the anisotroic displacement parameters of the six methyl C-atoms were restrained to be nearly isotropic. By the use of a *SAME* instruction, the bonds involving the C12', C13' and C14' were restrained to the same length as those involving the unprimed ones. In addition, the 1,3-related distances were restrained to be equal in both disordered moieties.

### Crystal data

**Table:**

C_24_H_33_NO_3_	*F*(000) = 832
*M**_r_* = 383.51	*D*_x_ = 1.142 Mg m^−^^3^
Monoclinic, *P*2_1_/*c*	Mo *K*α radiation, λ = 0.71073 Å
Hall symbol: -P 2ybc	Cell parameters from 1466 reflections
*a* = 17.788 (3) Å	θ = 2.1–21.3°
*b* = 8.9872 (14) Å	µ = 0.07 mm^−^^1^
*c* = 14.235 (2) Å	*T* = 100 K
β = 101.414 (2)°	Prism, colorless
*V* = 2230.7 (6) Å^3^	0.30 × 0.10 × 0.03 mm
*Z* = 4	

### Data collection

**Table:**

Bruker SMART APEX diffractometer	2428 reflections with *I* > 2σ(*I*)
Radiation source: fine-focus sealed tube	*R*_int_ = 0.092
graphite	θ_max_ = 25.0°, θ_min_ = 2.3°
ω scans	*h* = −20→21
17094 measured reflections	*k* = −10→10
3928 independent reflections	*l* = −16→16

### Refinement

**Table:**

Refinement on *F*^2^	Secondary atom site location: difference Fourier map
Least-squares matrix: full	Hydrogen site location: inferred from neighbouring sites
*R*[*F*^2^ > 2σ(*F*^2^)] = 0.058	H-atom parameters constrained
*wR*(*F*^2^) = 0.154	*w* = 1/[σ^2^(*F*_o_^2^) + (0.0662*P*)^2^ + 0.7154*P*] where *P* = (*F*_o_^2^ + 2*F*_c_^2^)/3
*S* = 1.03	(Δ/σ)_max_ = 0.001
3928 reflections	Δρ_max_ = 0.24 e Å^−^^3^
283 parameters	Δρ_min_ = −0.23 e Å^−^^3^
45 restraints	Extinction correction: *SHELXL97* (Sheldrick, 2008), Fc^*^=kFc[1+0.001xFc^2^λ^3^/sin(2θ)]^-1/4^
Primary atom site location: structure-invariant direct methods	Extinction coefficient: 0.0079 (12)

### Fractional atomic coordinates and isotropic or equivalent isotropic displacement parameters (Å^2^)

**Table:**

	*x*	*y*	*z*	*U*_iso_*/*U*_eq_	Occ. (<1)
O1	0.32463 (11)	0.7157 (2)	0.42898 (14)	0.0281 (5)	
H1O	0.3613	0.7145	0.3998	0.042*	
O2	0.46605 (11)	−0.2040 (2)	0.36814 (13)	0.0257 (5)	
O3	0.58429 (11)	−0.1107 (2)	0.41969 (13)	0.0237 (5)	
N1	0.41764 (13)	0.5020 (2)	0.31779 (16)	0.0223 (6)	
H1N	0.4497	0.5690	0.3479	0.027*	
C1	0.26841 (16)	0.6193 (3)	0.38515 (19)	0.0206 (6)	
C2	0.20301 (16)	0.6027 (3)	0.4259 (2)	0.0224 (7)	
C3	0.14725 (16)	0.5024 (3)	0.3809 (2)	0.0246 (7)	
H3	0.1028	0.4890	0.4076	0.029*	
C4	0.15296 (16)	0.4208 (3)	0.2993 (2)	0.0227 (7)	
C5	0.21844 (16)	0.4441 (3)	0.2617 (2)	0.0227 (7)	
H5	0.2236	0.3920	0.2052	0.027*	
C6	0.27635 (15)	0.5401 (3)	0.30322 (19)	0.0197 (6)	
C7	0.19398 (17)	0.6857 (3)	0.5172 (2)	0.0272 (7)	
C8	0.11681 (17)	0.6522 (4)	0.5457 (2)	0.0388 (8)	
H8A	0.1126	0.5451	0.5564	0.058*	
H8B	0.0748	0.6841	0.4942	0.058*	
H8C	0.1138	0.7061	0.6047	0.058*	
C9	0.25655 (17)	0.6366 (4)	0.6014 (2)	0.0332 (8)	
H9A	0.2537	0.5286	0.6097	0.050*	
H9B	0.2490	0.6867	0.6600	0.050*	
H9C	0.3070	0.6631	0.5885	0.050*	
C10	0.1981 (2)	0.8544 (3)	0.5028 (2)	0.0399 (9)	
H10A	0.2468	0.8797	0.4842	0.060*	
H10B	0.1949	0.9054	0.5627	0.060*	
H10C	0.1554	0.8860	0.4522	0.060*	
C11	0.09420 (17)	0.3034 (3)	0.2554 (2)	0.0365 (8)	
C12	0.0664 (3)	0.3407 (7)	0.1489 (3)	0.077 (2)	0.834 (6)
H12A	0.0275	0.2683	0.1199	0.115*	0.834 (6)
H12B	0.1099	0.3368	0.1161	0.115*	0.834 (6)
H12C	0.0442	0.4408	0.1428	0.115*	0.834 (6)
C13	0.1347 (3)	0.1529 (4)	0.2641 (4)	0.0600 (17)	0.834 (6)
H13A	0.0980	0.0750	0.2377	0.090*	0.834 (6)
H13B	0.1557	0.1318	0.3317	0.090*	0.834 (6)
H13C	0.1765	0.1552	0.2284	0.090*	0.834 (6)
C14	0.0263 (2)	0.2976 (5)	0.3027 (4)	0.0452 (13)	0.834 (6)
H14A	−0.0094	0.2209	0.2719	0.068*	0.834 (6)
H14B	0.0005	0.3944	0.2964	0.068*	0.834 (6)
H14C	0.0433	0.2735	0.3707	0.068*	0.834 (6)
C12'	0.1079 (13)	0.210 (2)	0.1736 (14)	0.048 (7)	0.166 (6)
H12D	0.1621	0.2163	0.1694	0.073*	0.166 (6)
H12E	0.0761	0.2472	0.1139	0.073*	0.166 (6)
H12F	0.0944	0.1068	0.1839	0.073*	0.166 (6)
C13'	0.0927 (15)	0.184 (2)	0.3315 (15)	0.074 (9)	0.166 (6)
H13D	0.1373	0.1185	0.3355	0.111*	0.166 (6)
H13E	0.0456	0.1248	0.3140	0.111*	0.166 (6)
H13F	0.0941	0.2313	0.3938	0.111*	0.166 (6)
C14'	0.0130 (7)	0.361 (2)	0.2415 (18)	0.048 (7)	0.166 (6)
H14D	−0.0041	0.3589	0.3028	0.072*	0.166 (6)
H14E	−0.0208	0.2991	0.1948	0.072*	0.166 (6)
H14F	0.0114	0.4641	0.2180	0.072*	0.166 (6)
C15	0.34660 (15)	0.5559 (3)	0.25896 (19)	0.0225 (7)	
H15A	0.3374	0.5013	0.1974	0.027*	
H15B	0.3531	0.6623	0.2445	0.027*	
C16	0.43888 (16)	0.3562 (3)	0.33031 (19)	0.0203 (6)	
C17	0.51327 (15)	0.3219 (3)	0.37876 (19)	0.0205 (6)	
H17	0.5482	0.4004	0.4004	0.025*	
C18	0.53670 (16)	0.1770 (3)	0.39565 (19)	0.0204 (6)	
H18	0.5876	0.1565	0.4282	0.024*	
C19	0.48608 (16)	0.0590 (3)	0.36514 (19)	0.0202 (6)	
C20	0.41230 (16)	0.0918 (3)	0.31703 (19)	0.0220 (7)	
H20	0.3774	0.0130	0.2960	0.026*	
C21	0.38877 (16)	0.2380 (3)	0.29915 (18)	0.0208 (6)	
H21	0.3382	0.2581	0.2654	0.025*	
C22	0.50943 (15)	−0.0962 (3)	0.38371 (18)	0.0194 (6)	
C23	0.61300 (17)	−0.2598 (3)	0.4479 (2)	0.0245 (7)	
H23A	0.6072	−0.3259	0.3912	0.029*	
H23B	0.5846	−0.3037	0.4944	0.029*	
C24	0.69625 (17)	−0.2403 (3)	0.4929 (2)	0.0320 (8)	
H24A	0.7188	−0.3374	0.5133	0.048*	
H24B	0.7009	−0.1744	0.5486	0.048*	
H24C	0.7234	−0.1964	0.4460	0.048*	

### Atomic displacement parameters (Å^2^)

**Table:**

	*U*^11^	*U*^22^	*U*^33^	*U*^12^	*U*^13^	*U*^23^
O1	0.0285 (12)	0.0281 (11)	0.0296 (12)	−0.0076 (9)	0.0102 (9)	−0.0064 (9)
O2	0.0288 (12)	0.0204 (11)	0.0285 (11)	−0.0036 (9)	0.0072 (9)	−0.0018 (9)
O3	0.0254 (11)	0.0184 (10)	0.0271 (11)	0.0027 (8)	0.0049 (9)	0.0050 (8)
N1	0.0229 (13)	0.0186 (13)	0.0255 (13)	0.0000 (10)	0.0045 (11)	−0.0029 (10)
C1	0.0232 (16)	0.0138 (14)	0.0229 (15)	0.0011 (12)	0.0001 (12)	−0.0007 (12)
C2	0.0201 (16)	0.0216 (15)	0.0254 (15)	0.0033 (12)	0.0044 (12)	0.0012 (12)
C3	0.0204 (16)	0.0246 (16)	0.0295 (17)	0.0006 (12)	0.0068 (13)	0.0009 (13)
C4	0.0191 (16)	0.0217 (15)	0.0252 (16)	0.0030 (12)	−0.0009 (13)	−0.0025 (12)
C5	0.0265 (17)	0.0197 (15)	0.0209 (15)	0.0051 (12)	0.0025 (13)	−0.0006 (12)
C6	0.0225 (16)	0.0176 (14)	0.0189 (14)	0.0036 (12)	0.0035 (12)	0.0029 (12)
C7	0.0275 (17)	0.0290 (17)	0.0277 (16)	0.0003 (13)	0.0114 (14)	−0.0037 (13)
C8	0.0319 (19)	0.051 (2)	0.0372 (19)	−0.0004 (16)	0.0151 (16)	−0.0107 (16)
C9	0.0332 (19)	0.0406 (19)	0.0275 (17)	−0.0082 (15)	0.0101 (14)	−0.0066 (15)
C10	0.047 (2)	0.0295 (18)	0.047 (2)	0.0008 (15)	0.0194 (17)	−0.0112 (16)
C11	0.0273 (18)	0.0364 (19)	0.044 (2)	−0.0023 (15)	0.0038 (16)	−0.0094 (16)
C12	0.063 (3)	0.113 (5)	0.047 (3)	−0.052 (3)	−0.009 (2)	−0.010 (3)
C13	0.045 (3)	0.033 (2)	0.106 (4)	−0.014 (2)	0.025 (3)	−0.032 (3)
C14	0.028 (2)	0.042 (3)	0.068 (3)	−0.0133 (19)	0.016 (2)	−0.016 (2)
C12'	0.043 (10)	0.047 (10)	0.053 (10)	−0.007 (8)	0.004 (8)	−0.026 (8)
C13'	0.078 (12)	0.063 (11)	0.078 (12)	−0.014 (9)	0.007 (9)	−0.005 (9)
C14'	0.046 (10)	0.050 (10)	0.048 (10)	−0.009 (8)	0.007 (8)	−0.012 (8)
C15	0.0259 (17)	0.0199 (15)	0.0218 (15)	0.0045 (12)	0.0047 (13)	0.0009 (12)
C16	0.0259 (16)	0.0195 (15)	0.0174 (14)	0.0004 (12)	0.0090 (12)	−0.0002 (12)
C17	0.0234 (16)	0.0173 (14)	0.0220 (15)	−0.0040 (12)	0.0075 (13)	−0.0046 (12)
C18	0.0201 (15)	0.0225 (15)	0.0191 (14)	0.0006 (12)	0.0054 (12)	−0.0018 (12)
C19	0.0218 (16)	0.0220 (15)	0.0181 (14)	0.0009 (12)	0.0072 (12)	−0.0004 (12)
C20	0.0244 (16)	0.0193 (15)	0.0234 (15)	−0.0033 (12)	0.0075 (13)	−0.0012 (12)
C21	0.0209 (15)	0.0245 (16)	0.0177 (15)	0.0000 (12)	0.0058 (12)	−0.0026 (12)
C22	0.0193 (16)	0.0228 (15)	0.0176 (14)	−0.0010 (13)	0.0070 (12)	−0.0016 (12)
C23	0.0359 (18)	0.0181 (15)	0.0212 (15)	0.0056 (13)	0.0095 (13)	0.0042 (12)
C24	0.0344 (19)	0.0292 (17)	0.0323 (18)	0.0076 (14)	0.0065 (15)	0.0098 (14)

### Geometric parameters (Å, °)

**Table:**

O1—C1	1.376 (3)	C12—H12A	0.9800
O1—H1O	0.8400	C12—H12B	0.9800
O2—C22	1.231 (3)	C12—H12C	0.9800
O3—C22	1.335 (3)	C13—H13A	0.9800
O3—C23	1.462 (3)	C13—H13B	0.9800
N1—C16	1.366 (3)	C13—H13C	0.9800
N1—C15	1.454 (3)	C14—H14A	0.9800
N1—H1N	0.8800	C14—H14B	0.9800
C1—C6	1.397 (4)	C14—H14C	0.9800
C1—C2	1.407 (4)	C12'—H12D	0.9800
C2—C3	1.398 (4)	C12'—H12E	0.9800
C2—C7	1.534 (4)	C12'—H12F	0.9800
C3—C4	1.395 (4)	C13'—H13D	0.9800
C3—H3	0.9500	C13'—H13E	0.9800
C4—C5	1.391 (4)	C13'—H13F	0.9800
C4—C11	1.529 (4)	C14'—H14D	0.9800
C5—C6	1.384 (4)	C14'—H14E	0.9800
C5—H5	0.9500	C14'—H14F	0.9800
C6—C15	1.513 (4)	C15—H15A	0.9900
C7—C10	1.534 (4)	C15—H15B	0.9900
C7—C9	1.531 (4)	C16—C17	1.400 (4)
C7—C8	1.536 (4)	C16—C21	1.401 (4)
C8—H8A	0.9800	C17—C18	1.374 (4)
C8—H8B	0.9800	C17—H17	0.9500
C8—H8C	0.9800	C18—C19	1.403 (4)
C9—H9A	0.9800	C18—H18	0.9500
C9—H9B	0.9800	C19—C20	1.387 (4)
C9—H9C	0.9800	C19—C22	1.465 (4)
C10—H10A	0.9800	C20—C21	1.387 (4)
C10—H10B	0.9800	C20—H20	0.9500
C10—H10C	0.9800	C21—H21	0.9500
C11—C12'	1.491 (9)	C23—C24	1.503 (4)
C11—C14	1.495 (5)	C23—H23A	0.9900
C11—C14'	1.511 (9)	C23—H23B	0.9900
C11—C13	1.526 (5)	C24—H24A	0.9800
C11—C13'	1.531 (10)	C24—H24B	0.9800
C11—C12	1.535 (5)	C24—H24C	0.9800
			
C1—O1—H1O	109.5	H13A—C13—H13B	109.5
C22—O3—C23	117.5 (2)	C11—C13—H13C	109.5
C16—N1—C15	125.5 (2)	H13A—C13—H13C	109.5
C16—N1—H1N	117.2	H13B—C13—H13C	109.5
C15—N1—H1N	117.2	C11—C14—H14A	109.5
O1—C1—C6	121.0 (3)	C11—C14—H14B	109.5
O1—C1—C2	117.8 (2)	H14A—C14—H14B	109.5
C6—C1—C2	121.2 (2)	C11—C14—H14C	109.5
C3—C2—C1	116.7 (3)	H14A—C14—H14C	109.5
C3—C2—C7	121.1 (3)	H14B—C14—H14C	109.5
C1—C2—C7	122.1 (2)	C11—C12'—H12D	109.5
C4—C3—C2	123.9 (3)	C11—C12'—H12E	109.5
C4—C3—H3	118.0	H12D—C12'—H12E	109.5
C2—C3—H3	118.0	C11—C12'—H12F	109.5
C5—C4—C3	116.5 (2)	H12D—C12'—H12F	109.5
C5—C4—C11	120.2 (3)	H12E—C12'—H12F	109.5
C3—C4—C11	123.2 (3)	C11—C13'—H13D	109.5
C6—C5—C4	122.6 (3)	C11—C13'—H13E	109.5
C6—C5—H5	118.7	H13D—C13'—H13E	109.5
C4—C5—H5	118.7	C11—C13'—H13F	109.5
C5—C6—C1	119.0 (3)	H13D—C13'—H13F	109.5
C5—C6—C15	119.1 (2)	H13E—C13'—H13F	109.5
C1—C6—C15	121.9 (2)	C11—C14'—H14D	109.5
C10—C7—C9	110.0 (3)	C11—C14'—H14E	109.5
C10—C7—C8	107.4 (3)	H14D—C14'—H14E	109.5
C9—C7—C8	106.6 (2)	C11—C14'—H14F	109.5
C10—C7—C2	110.6 (2)	H14D—C14'—H14F	109.5
C9—C7—C2	110.0 (2)	H14E—C14'—H14F	109.5
C8—C7—C2	112.2 (2)	N1—C15—C6	115.1 (2)
C7—C8—H8A	109.5	N1—C15—H15A	108.5
C7—C8—H8B	109.5	C6—C15—H15A	108.5
H8A—C8—H8B	109.5	N1—C15—H15B	108.5
C7—C8—H8C	109.5	C6—C15—H15B	108.5
H8A—C8—H8C	109.5	H15A—C15—H15B	107.5
H8B—C8—H8C	109.5	N1—C16—C17	119.0 (2)
C7—C9—H9A	109.5	N1—C16—C21	123.0 (2)
C7—C9—H9B	109.5	C17—C16—C21	118.0 (2)
H9A—C9—H9B	109.5	C18—C17—C16	121.3 (2)
C7—C9—H9C	109.5	C18—C17—H17	119.4
H9A—C9—H9C	109.5	C16—C17—H17	119.4
H9B—C9—H9C	109.5	C17—C18—C19	120.6 (3)
C7—C10—H10A	109.5	C17—C18—H18	119.7
C7—C10—H10B	109.5	C19—C18—H18	119.7
H10A—C10—H10B	109.5	C20—C19—C18	118.6 (2)
C7—C10—H10C	109.5	C20—C19—C22	119.9 (2)
H10A—C10—H10C	109.5	C18—C19—C22	121.5 (2)
H10B—C10—H10C	109.5	C19—C20—C21	120.9 (3)
C12'—C11—C14'	113.0 (13)	C19—C20—H20	119.5
C14—C11—C13	110.1 (3)	C21—C20—H20	119.5
C12'—C11—C13'	100.8 (15)	C20—C21—C16	120.7 (3)
C14'—C11—C13'	100.6 (13)	C20—C21—H21	119.7
C12'—C11—C4	120.1 (9)	C16—C21—H21	119.7
C14—C11—C4	112.8 (3)	O2—C22—O3	122.3 (2)
C14'—C11—C4	111.9 (9)	O2—C22—C19	124.9 (2)
C13—C11—C4	107.7 (3)	O3—C22—C19	112.8 (2)
C13'—C11—C4	107.6 (10)	O3—C23—C24	105.7 (2)
C14—C11—C12	108.7 (3)	O3—C23—H23A	110.6
C13—C11—C12	109.1 (4)	C24—C23—H23A	110.6
C4—C11—C12	108.3 (3)	O3—C23—H23B	110.6
C11—C12—H12A	109.5	C24—C23—H23B	110.6
C11—C12—H12B	109.5	H23A—C23—H23B	108.7
H12A—C12—H12B	109.5	C23—C24—H24A	109.5
C11—C12—H12C	109.5	C23—C24—H24B	109.5
H12A—C12—H12C	109.5	H24A—C24—H24B	109.5
H12B—C12—H12C	109.5	C23—C24—H24C	109.5
C11—C13—H13A	109.5	H24A—C24—H24C	109.5
C11—C13—H13B	109.5	H24B—C24—H24C	109.5
			
O1—C1—C2—C3	−179.0 (2)	C5—C4—C11—C13	60.4 (4)
C6—C1—C2—C3	0.7 (4)	C3—C4—C11—C13	−115.5 (4)
O1—C1—C2—C7	−1.2 (4)	C5—C4—C11—C13'	116.0 (12)
C6—C1—C2—C7	178.5 (2)	C3—C4—C11—C13'	−59.9 (12)
C1—C2—C3—C4	−0.5 (4)	C5—C4—C11—C12	−57.5 (4)
C7—C2—C3—C4	−178.3 (3)	C3—C4—C11—C12	126.6 (4)
C2—C3—C4—C5	−0.6 (4)	C16—N1—C15—C6	77.4 (3)
C2—C3—C4—C11	175.5 (3)	C5—C6—C15—N1	−113.9 (3)
C3—C4—C5—C6	1.5 (4)	C1—C6—C15—N1	65.8 (3)
C11—C4—C5—C6	−174.7 (3)	C15—N1—C16—C17	171.6 (2)
C4—C5—C6—C1	−1.3 (4)	C15—N1—C16—C21	−10.2 (4)
C4—C5—C6—C15	178.4 (2)	N1—C16—C17—C18	178.1 (3)
O1—C1—C6—C5	179.8 (2)	C21—C16—C17—C18	−0.1 (4)
C2—C1—C6—C5	0.2 (4)	C16—C17—C18—C19	−0.5 (4)
O1—C1—C6—C15	0.1 (4)	C17—C18—C19—C20	0.5 (4)
C2—C1—C6—C15	−179.6 (2)	C17—C18—C19—C22	−179.0 (2)
C3—C2—C7—C10	−123.6 (3)	C18—C19—C20—C21	0.1 (4)
C1—C2—C7—C10	58.7 (3)	C22—C19—C20—C21	179.6 (3)
C3—C2—C7—C9	114.7 (3)	C19—C20—C21—C16	−0.7 (4)
C1—C2—C7—C9	−63.1 (3)	N1—C16—C21—C20	−177.4 (3)
C3—C2—C7—C8	−3.8 (4)	C17—C16—C21—C20	0.7 (4)
C1—C2—C7—C8	178.5 (3)	C23—O3—C22—O2	−4.9 (4)
C5—C4—C11—C12'	1.7 (13)	C23—O3—C22—C19	175.4 (2)
C3—C4—C11—C12'	−174.2 (12)	C20—C19—C22—O2	−7.5 (4)
C5—C4—C11—C14	−177.9 (3)	C18—C19—C22—O2	172.0 (3)
C3—C4—C11—C14	6.2 (4)	C20—C19—C22—O3	172.2 (2)
C5—C4—C11—C14'	−134.4 (11)	C18—C19—C22—O3	−8.3 (4)
C3—C4—C11—C14'	49.7 (11)	C22—O3—C23—C24	−175.3 (2)

### Hydrogen-bond geometry (Å, °)

**Table:**

*D*—H···*A*	*D*—H	H···*A*	*D*···*A*	*D*—H···*A*
O1—H1o···O2^i^	0.84	2.13	2.909 (3)	154
N1—H1n···O2^i^	0.88	2.07	2.827 (3)	143

Symmetry codes: (i) *x*, *y*+1, *z*.
